# Effect of Herb-Partitioned Moxibustion on Autophagy and Immune-Associated Gene Expression Profiles in a Rat Model of Crohn's Disease

**DOI:** 10.1155/2019/3405146

**Published:** 2019-03-07

**Authors:** Ji-meng Zhao, Ya-nan Liu, Han-dan Zheng, Yan Huang, Qin Qi, Hui-rong Liu, Yin Shi, Xiao-peng Ma, Yuan Lu, Lu-yi Wu

**Affiliations:** ^1^Key Laboratory of Acupuncture and Immunological Effects, Shanghai Research Institute of Acupuncture and Meridian, Shanghai University of Traditional Chinese Medicine, Shanghai 200030, China; ^2^Shanghai University of Traditional Chinese Medicine, Shanghai 201203, China; ^3^Shanghai Qigong Research Institute, Shanghai University of Traditional Chinese Medicine, Shanghai 200030, China

## Abstract

**Objective:**

To investigate the immune regulation mechanism of herb-partitioned moxibustion in rats with Crohn's disease (CD) focusing on autophagy.

**Methods:**

Rats were randomly divided into normal (N) group, CD model (M) group, CD model with herb-partitioned moxibustion (MM) group, normal with herb-partitioned moxibustion (NM) group, CD model with mesalazine (western medicine, Med ) group, and normal saline (NS) group, with 10 rats in each group. The CD model rats were prepared by trinitrobenzene sulphonic expect for the N group and NM group. After the CD rats model were established, the rats in the MM and NM groups were treated with herb-partitioned moxibustion at Tianshu (ST25) and Qihai (CV6) acupoints once daily for 7 days, and rats in the Med and NS groups were respectively treated with mesalazine enteric coated tablet and normal saline once daily for 7 days. After intervention, hematoxylin-eosin staining was used to observe the histological changes of colon; RNA sequencing was used to observe the changes in autophagy- and immune-associated gene expression profiles. In addition, autophagy- and immune-associated cytokines and signaling pathways in CD rats were also screened.

**Results:**

HPM significantly increased the body weight of CD rats (*P*<0.01) and improved the pathological injury of colon in CD rats (*P*<0.01). HPM also changed the expression of many autophagy- and immune-associated genes, especially downregulating the expression of autophagy-associated* Nod2*,* Irgm* genes as well as the receptor of immune-associated* Il12b*,* Il22 *(*Il12rb1*,* Il22ra2*) genes in the colon of CD rats. HPM also changed the enrichment levels of differentially expressed genes in the human T-cell leukemia virus type-1 infection pathway, the Epstein-Barr virus infection pathway, and the cell adhesion molecule pathway. In addition, the expression levels of Nod2, Irgm, IL-12b, and IL-22 mRNA were increased (all* P*< 0.01) in the M group compared to the N group, while the expression levels of Nod2, Irgm, IL-12b, and IL-22 mRNA were decreased (*P*<0.05 or* P*<0.01) in the MM and Med groups compared to the M group.

**Conclusion:**

Herb-partitioned moxibustion may effectively attenuate intestinal inflammation and promote the repair of colon mucosal injury of CD rats through the regulation of autophagy- and immune-associated gene expression and signaling pathways.

## 1. Introduction

Crohn's disease (CD) is a chronic, nonspecific inflammatory bowel disease (IBD) with unknown etiology and it can affect any part of the gastrointestinal tract from mouth to anus but mainly develops in the colon and the terminal ileum. Its main clinical manifestations are abdominal pain, diarrhea, and bloody purulent stool. The pathogenesis of CD is not completely understood. Most studies suggest that the occurrence of CD is associated with genetics, immunity, environment, and intestinal microecology. As one type of cellular stress response, autophagy plays a critical role in innate immunity and adaptive immunity and is an important component of immune homeostasis. The autophagy pathway and biosynthesis together maintain the dynamic equilibrium of intracellular macromolecules [1] that play important roles in the development and progression of diseases. In recent years, the function of autophagy and some autophagy-associated genes in the development of IBD has been confirmed. Autophagy plays multiple roles in the pathogenesis of IBD by altering intracellular bactericidal effects, goblet cell function, proinflammatory cytokine production by macrophages, antigen presentation of dendritic cell, and endoplasmic reticulum stress response in intestinal cells [2].

Our previous studies indicated that herb-partitioned moxibustion (HPM) had good effect on the treatment of CD [3–6]; however, the mechanism of its effect requires further exploration. Therefore, the investigation of the pathogenic mechanism of CD and the immune-regulatory mechanism of HPM on CD with a focus on autophagy will be important in the study of pathogenesis, diagnosis, and treatment of CD. The rapid development of transcriptomics, proteomics, and metabolomics provides new methods for investigating the mechanisms of acupuncture and moxibustion. The transcriptome is the sum of all RNAs transcribed in a developmental stage or functional status of specific tissues or cells. Transcriptomics can be used to study gene expression, structure, and function at a global level to reveal the molecular mechanism of specific biological or pathological processes. As a next-generation transcriptome sequencing technology, RNA sequencing (RNA-Seq) has the advantages of high throughput, high sensitivity, high resolution, and not being subject to sample restrictions, which can provide reliable technical support for studying gene structure and function. Therefore, our study used RNA-Seq to observe changes of autophagy- and immune-associated gene expression profiles in colon tissues of CD rats and the regulatory effect of HPM. It will provide new ideas for understanding the development of CD and the mechanism of HPM treatment from the perspective of autophagy.

## 2. Materials and Methods

### 2.1. Experimental Animals

Adult male Sprague-Dawley (SD) rats, weighing 180±20g, were provided by the Experimental Animal Center of Shanghai University of Traditional Chinese Medicine and were purchased from Vital River Laboratory Animal Technology Co., Ltd. (Beijing, China). The license for the use of experimental animals was SCXK (Beijing) 2012-0001. All the animals were housed in a clean grade room with controlled temperature (20±2°C), a light/dark (12 h: 12 h) cycle, and 50–70% indoor humidity. All experimental protocols were approved by the Animal Research Ethics Committee of Shanghai University of Traditional Chinese Medicine.

### 2.2. Establishment of CD Model

After one week of adaptive feeding, the 60 rats were randomly divided into normal (N), CD model (M), CD model with herb-partitioned moxibustion (MM), normal with herb-partitioned moxibustion (NM), CD model with mesalazine (western medicine, Med) group, and CD model with normal saline (NS) groups, with 10 rats in each group. The CD models were established by 2,4,6-trinitro-benzene-sulfonic acid (TNBS, Sigma, USA) according to Morris' method [7] except for the N and NM groups. The rats were fasted and given water for 24h, then rats were weighed and 2% sodium pentobarbital (30 mg/kg) was used for anesthesia through intraperitoneal injection. The rats were anally injected with 5% TNBS solution mixed in 50% alcohol at a 1:2 ratio (3ml/kg) using a rubber tube, with a deep of 6-8 cm, and the head of the rat was pushed down for about 1 min to prevent loss of the injected solution. The injection was repeated every 7 days for 4 weeks. After the CD rat models were established, HE staining was used to observe whether the CD model was successfully established.

### 2.3. Moxibustion Intervention

After the experimental CD rat models were successfully established, the rats were exposed to different treatments. In the MM and NM groups, the Tianshu (bilateral, ST25) and Qihai (CV6) acupoints were selected [8–10]. The herbal cake was Chinese medicine powder (Coptis chinensis, Radix aconiti lateralis, Cortex Cinnamomi, Radix aucklandiae, Flos carthami, Salvia miltiorrhiza, and Angelica sinensis) mixed and stirred with yellow wine to form a thick paste; the herbal cake was prepared with 1 cm in diameter and 0.45cm in thickness using a mold. The moxa cone was made of about 90 mg using a mold. HPM treatment was performed with the moxa cone placed on the top of the herbal cake at the ST25 and CV6 acupoints and ignited. Two moxa cones were used at each acupoint for each treatment once daily for 7 days. The rats in the Med group were fed with mesalazine (Losan Pharma GmbH, Germany), which was prepared at the proportion of adult and rat of 1:0.018 [11], once a day for 7 days. The rats in the NS group were fed with normal saline, 2 ml per time and once a day for 7 days. The rats in the N and M groups did not receive any treatment but were grabbed and immobilized using the same method applied to other groups.

### 2.4. Sample Collection

Rats were anesthetized by intraperitoneal injection of 1% pentobarbital sodium (30 mg/kg). After anesthetization, the abdominal cavity was opened and 4-6 cm of distal colon was collected 1 cm from the anus. The colon was divided into three parts, one part was fixed in 10% neutral formalin fixative solution, and the other two parts were placed in cryotubes after cutting into pieces, then frozen in liquid nitrogen for 1 h, and later stored in a -80°C freezer.

### 2.5. Histological Observation and Microscopic Scoring

Rat colon tissues were fixed in 10% neutral formalin fixative solution for 24 h, dehydrated, embedded in paraffin, sectioned at a thickness of 4 *μ*m, and subjected to HE staining. Histological changes of the colon tissues were observed under an optical microscope (BX33, Olympus) and scoring of pathological injury was performed according to the Score Criteria of Colonic Histological Damage [12].

### 2.6. RNA Extraction and Quality Control

Two colon tissue samples were randomly selected from each group, and total RNA was extracted using the Trizol (Bio-Rad, USA) method. The purity, concentration, and integrity of the total RNA were determined. Quality control of the total RNA was performed using the Agilent 2100 Bioanalyzer. Samples with an RNA integrity number higher than 8 and a concentration ≥500ng/*μ*L were used for library construction and RNA-Seq.

### 2.7. Construction of the RNA Library

A certain amount of the total RNA was collected, and the DNA fragments present in the total RNA sample were digested using DNase I. mRNA was purified using oligo (dT) magnetic beads (Invitrogen, USA) and then eluted using a buffer solution and placed on ice. A breaking agent was added, and the mixture reacted in a Thermomixer at an appropriate temperature for the specified time. The sample was precipitated, and the broken product was recovered. The broken mRNA was mixed thoroughly with an appropriate amount of primers, and the mixture was placed in a Thermomixer at an appropriate temperature for a certain amount of time to allow for secondary structure opening for primer binding. The preprepared first-chain synthesis reaction sample was added, and the first-chain cDNA was synthesized in a PCR machine using the corresponding program. Second-chain cDNA was also synthesized using the same method. After end repair, the sample was dissolved in EB solution. Next, an adenine was added to the cDNA 3' end, and an adaptor was ligated to the cDNA 5' end. The ligated product was subject to agarose gel electrophoresis. The gel was cut according to the size of the DNA fragment. The PCR sample was prepared, and an appropriate PCR program was selected to amplify the obtained ligated product to complete the construction of RNA library.

### 2.8. RNA Sequencing and Data Analysis

The quality of the RNA library was detected using the Agilent 2100 and ABI Step One Plus Real-Time PCR System, then the samples were processed and sequenced using the Hiseq400 sequencing platform. Data collection software provided by Illumina was used to control the sequencing process and perform real-time data analysis. For the comparison between two groups, differentially expressed genes were defined as genes with a fold change (FC) ≥1.5. The log_2_ ratio value was the normalized log_2_ FC value, i.e., |log_2_ ratio| ≥ 0.58. For example, a log_2_ ratio value of group A/group B higher than 0.58 indicated that the gene was upregulated in group A compared to that in the group B and a log_2_ ratio value lower than -0.58 indicated that the gene was downregulated in group A compared to that in the group B.

### 2.9. Validation of Differentially Expressed Gene Using qRT-PCR.

Total RNA extraction was performed using Trizol method, and the selected differentially expressed genes were validated through reverse transcription and real-time PCR amplification. The 2^−ΔΔCt^ method was performed to analyze relative mRNA expression in samples of colon tissue.

### 2.10. Statistical Analysis

The SPSS 18.0 software was used for the data analysis. The data were presented as the mean ± standard deviation ($\overset{-}{x}\pm s$) and the comparison of differences among groups was performed using one-way ANOVA if data conformed to the normal distribution. If the variances were homogeneous, the pairwise comparison was performed using the least significant difference (LSD) test, and Dunnett's T3 method was performed if the variances were not homogenous. Data that did not conform to the normal distribution were presented as the median and quartiles [M(P25,P75)] and Nonparametric Kruskal Wallis test was performed for the comparison of differences among groups. The significance level of the statistical examination was *α*=0.05.* P*< 0.05 indicated that the difference had statistical significance.

## 3. Results

### 3.1. Changes of the Body Weights of Rats

The body weights of the rats had no significant differences among the groups before the model establishment. After the CD model was established, the body weights of the rats in the M group, MM group, Med group, and NS group were significantly decreased compared with the N group (*P*<0.01 or* P*<0.05), while there was no significant difference between the N group and the NM group rats; the body weights of the rats in the M group, MM group, and NS group were also significantly decreased compared with the NM group (*P*<0.01 or* P*<0.05). After the treatment, the body weights of the rats in the M group were still significantly decreased compared with the N group (*P*<0.01); the body weights of the rats in the MM group, Med group, and NS group were significantly increased compared with the M group (*P*<0.01); there was also no significant difference between the N group and the NM group rats (Supplementary Figure S1).

### 3.2. Histological Changes and the Injury Scores of Colon Tissues

**Figure 1:**
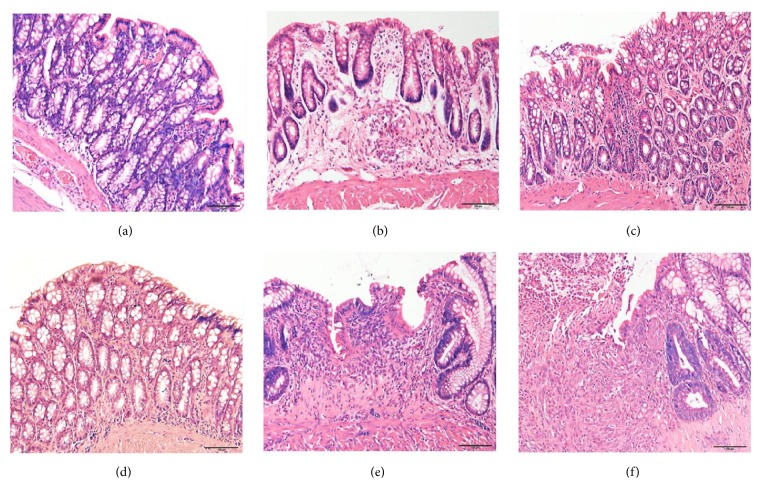
The histological observation of rat colon in each group by HE staining method, (200×). (a) Normal group;(b) CD model group; (c) CD model with herb-partitioned moxibustion group; (d) Normal with herb-partitioned moxibustion group; (e) CD model with mesalazine group; (f) CD model with normal saline group.

In the N and NM group, the epithelial structure of the colon tissues was clear, with intact and continuous colon mucosa, the arrangement of glands was ordered, a small number of inflammatory cells were observed, and there was no hyperemia, edema, hyperplasia, or ulcer appeared. In the M group, monocyte-macrophage hyperplasia was observed in the submucosal layer of the colon tissues, with dilation, hyperemia, edema of the surrounding blood vessel, disordered tissue structure, gland dilation, and granuloma formation. In the MM group, the mucosal epithelium of the colon tissues was more intact than the M group, some glands were lost with heal shallow ulcers, and there was a few inflammatory cells and fibrocytes infiltration. In the Med group, the colon mucosal epithelial was covered by hyperplasia tissues, formation of small healing ulcers, atypical hyperplasia of glands on the ulcer edge, a large number of fibroblasts in the submucosal layer, and hyperplasia of new capillaries. In the NS group, a large amount of mucosa was lost and presented as giant ulcers that reach the muscular layer, atypical hyperplasia of gland epithelial cells, cell exudation and necrosis on the surface of ulcers, and a large number of granulomas at the bottom of ulcers (Figure 1).

**Figure 2:**
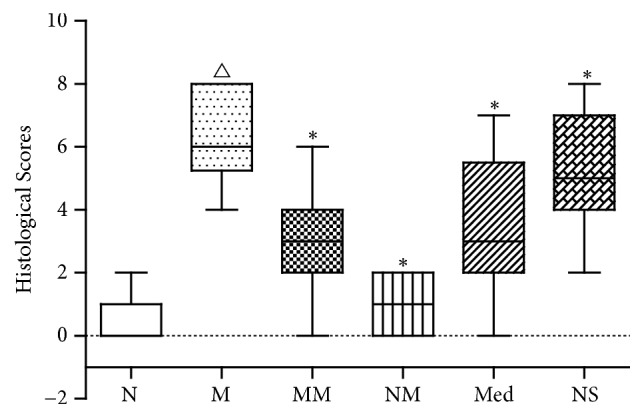
Histological scores of rat colon in each group. N: normal group; M: CD model group; MM: CD model with herb-partitioned moxibustion group; NM: normal with herb-partitioned moxibustion group; Med: CD model with mesalazine group; NS: CD model with normal saline group. ^△^*P*<0.01 versus normal group; ^*∗*^*P*<0.01 versus model group. P values between different groups were calculated from nonparametric test.

The pathological injury scores showed that the colon injury scores in the M group were significantly higher than those of the normal group, and the difference was statistically significant. While the colon injury scores in the MM and Med groups were both significantly decreased compared to the M group (Figure 2).

### 3.3. Correlation Analyses of Gene Expression between Samples

**Figure 3:**
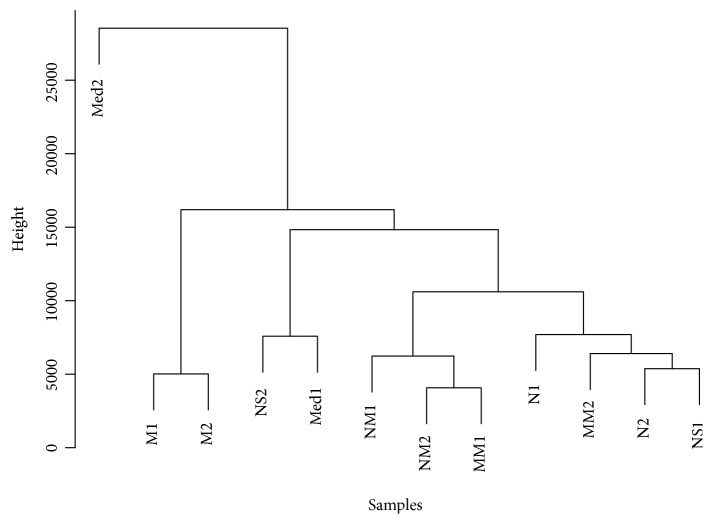
Clustering tree of distances between different groups. N: normal group; M: CD model group; MM: CD model with herb-partitioned moxibustion group; NM: normal with herb-partitioned moxibustion group; Med: CD model with mesalazine group; NS: CD model with normal saline group.

The Euclidean distance calculation method was used to calculate the distance of gene expression levels in all samples. As shown in Figure 3, the vertical axis indicates the height of the clustering tree. Samples with similar heights were prone to cluster together. The distance between samples was also calculated using the square sum of deviations method (Ward's method). A cluster plot was established to represent the distances between samples, which intuitively reflected the difference between samples. Our result shows that the CD model group and all the treatment groups generally belonged to two classes. The difference between the CD model group and the N group was significant, while the difference among the MM group, NM group, and the N group was smaller.

In addition, we performed heatmap analyses on the correlation between samples (Supplementary Figure S2). When the correlation value was smaller (closer to 0), the color was close to white; when the correlation value was larger (closer to 1), the color was close to blue. It showed that the difference between NM1 and Med2 was largest. Overall, the differences between the N group and the M group as well as the NM group and the M group were larger, while the differences between the Med group and the M group as well as the NS group and the M group were smaller.

### 3.4. Analysis of Significantly Differential Genes between Groups

Based on the whole gene expression of the RNA-Seq, the comparison of gene expression between different groups was observed. The comparison between the normal group and the CD model group showed 42 upregulated genes and 39 downregulated genes. The comparison between the MM group and the CD model group showed 93 upregulated genes and 93 downregulated genes. The comparison between the NM group and CD model group showed 126 upregulated genes and 158 downregulated genes. The comparison between the Med group and CD model group showed 57 upregulated genes and 83 downregulated genes. The comparison between the NS group and CD model group showed 80 upregulated genes and 41 downregulated genes. The comparison between the MM group and Med group showed 110 upregulated genes and 86 downregulated genes (Supplementary Figure S3).

**Figure 4:**
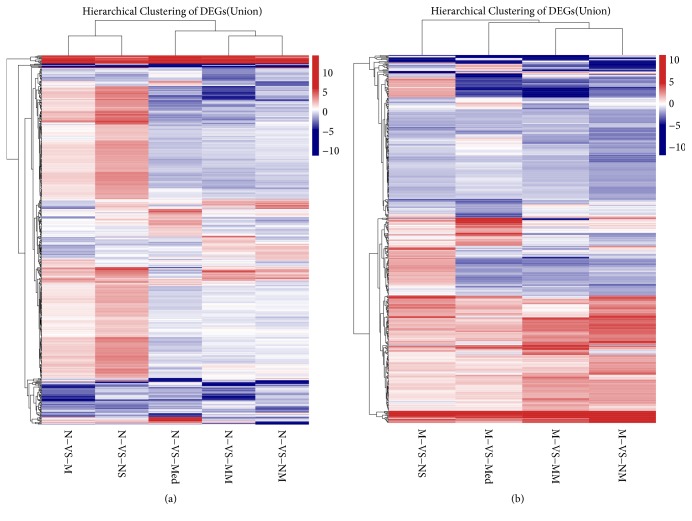
Heatmap of the clustering expression profiles of differential genes in all groups. (a) Comparison of differential gene expression between normal group and other groups. (b) Comparison of differential gene expression between CD model group and other groups. The gradient map indicates the differential fold values after logarithmic conversion. Each column indicates one differential pair, and each row indicates one differential gene. Different types of differential genes are represented by different colors: red indicates upregulation and blue indicates downregulation. The levels of upregulation and downregulation increased with the increasing darkness of the color. N: normal group; M: CD model group; MM: CD model with herb-partitioned moxibustion group; NM: normal with herb-partitioned moxibustion group; Med: CD model with mesalazine group; NS: CD model with normal saline group.

Next, cluster analyses were performed on significantly differentially expressed genes (Figure 4). The differential genes in the MM group, the NM group, and the Med group had low levels of upregulation or downregulation compared to the N group, indicating similar gene expression between each of these groups and the N group. While the upregulation of genes in the NS group was significant compared to that in the normal group, the differences of gene expression between the NS group and NC group were larger. The differential genes in the MM group had high levels of upregulation or downregulation with respect to the M group, indicating that there were large differences of gene expression between the MM group and the M group. Compared to the M group, the overall colors of differential genes were relatively lighter in the Med group and the NS group, suggesting that the levels of upregulation and downregulation of differential genes in the Med group and the NS group were lower with respect to the M group.

### 3.5. Analysis of Differential Gene Expression Profiles of Autophagy- and Immune-Associated Molecules between Groups

We further calculated the gene expression level of each sample and the expression levels of detected genes between different groups were compared. The differentially expressed genes were defined as those with a difference ≥1.5-fold change. Compared to the N group, the M group had 11 genes that were upregulated and two genes that were downregulated in which the CD autophagy-associated gene* Nod2 *was upregulated 1.357-fold,* Irgm* was upregulated 1.106-fold, and* Atg9b* was upregulated 2.459-fold; the immune-associated cytokine gene* Il12b* was upregulated 6.119-fold,* Il22* was upregulated 3.858-fold, and* Il23r* was downregulated 1.322-fold (Table 1). Compared to M group, the MM group had five genes that were upregulated and eight genes that were downregulated in which* Nod2* was downregulated 0.902-fold,* Irgm2* was downregulated 0.68-fold, and* Atg9b* was downregulated 2.459-fold; the immune-associated cytokine gene* Il22ra2* was downregulated 2.324-fold,* Il22ra1* was downregulated 0.613-fold, and* Il12rb1* was downregulated 1.067-fold (Table 2). Compared to the M group, the Med group had four genes that were upregulated and 15 genes that were downregulated in which* Nod2* was downregulated 0.771-fold,* Irgm* was downregulated 0.84-fold, the immune-associated cytokine gene* Il22* was downregulated 3.858-fold, and Il12b was downregulated 3.119-fold, while* Tgfb1* and* Tgfb2*of, the TGF family, were downregulated 1.548-fold and 0.996-fold, respectively (Table 3). Compared to the M group, the NS group had eight genes that were upregulated and 10 genes that were downregulated, in which* Atg9b* was downregulated 0.874-fold, the immune-associated cytokine gene* Ifng* was upregulated 5.426-fold,* Il27ra* was upregulated 2.015-fold, and* Il21* was upregulated 1.216-fold (Table 4).

### 3.6. Pathway Enrichment of Differential Genes

**Figure 5:**
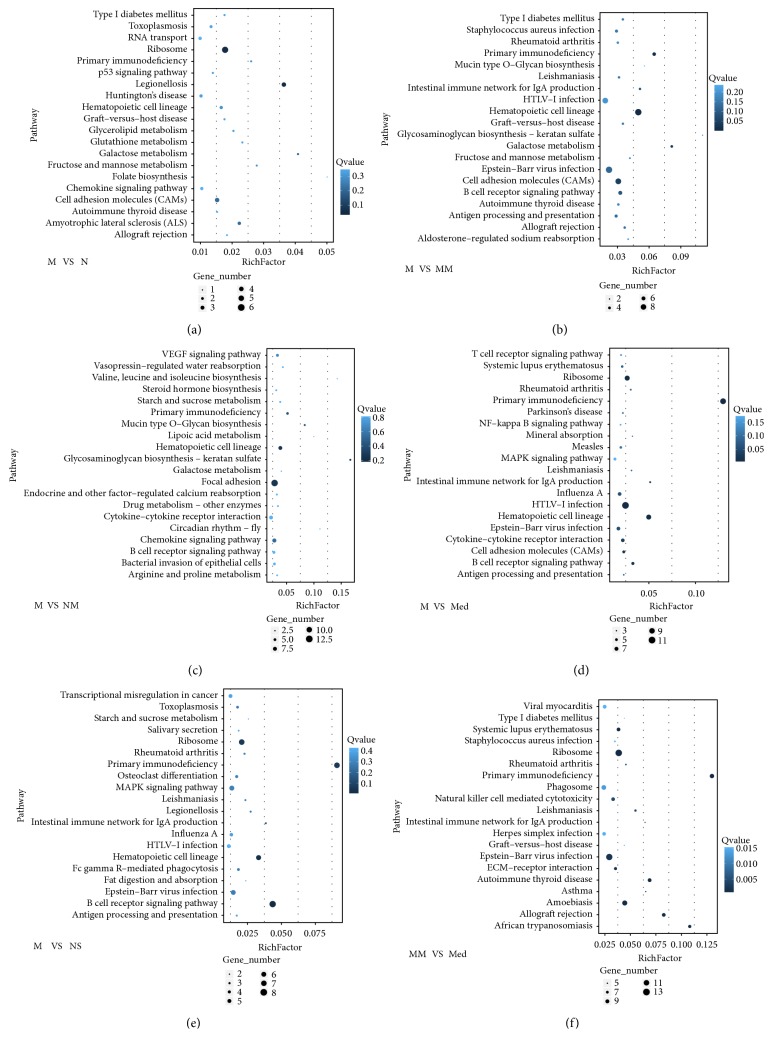
The scatter plot of pathway enrichments of differential genes between groups. (a) M group versus N group. (b) M group versus MM group. (c) M group versus NM group. (d) M group versus Med group. (e) M group versus NS group. (f) MM group versus Med group. The enrichment factor refers to the ratio between the number of differentially expressed genes in the pathway item and the total number of annotated genes in the same pathway item. When the enrichment factor was larger, the enrichment level was higher. The Q value was the normalized P value after multiple hypothesis testing, and the range of values was 0 to 1. When the value was close to 0, the enrichment was more significant. The figure only shows the pathway items with the top 20 enrichment levels. N: normal group; M: CD model group; MM: CD model with herb-partitioned moxibustion group; NM: normal with herb-partitioned moxibustion group; Med: CD model with mesalazine group; NS: CD model with normal saline group.

The pathway enrichment results for differential genes (Figure 5) showed that the pathways with higher enrichment levels between the N group and the M group included the cell adhesion molecule (CAM) pathway and the Ribosome pathway. The pathways with higher enrichment levels between the MM group and the model group included HTLV-1 infection pathway, Epstein-Barr virus infection, and CAM pathway. While the pathways with higher enrichment levels between the Med group and the model group included MAPK signaling pathway, B cell receptor signaling pathway, the pathways with higher enrichment levels between the NS group and the model group included MAPK signaling pathway, HTLV-1 infection pathway, Ribosome pathway, the pathways with higher enrichment levels between the MM group and the Med group included Ribosome pathway, Phagosome pathway.

### 3.7. Validation of Differential Expression mRNA in Colon Tissues of Rats

**Figure 6:**
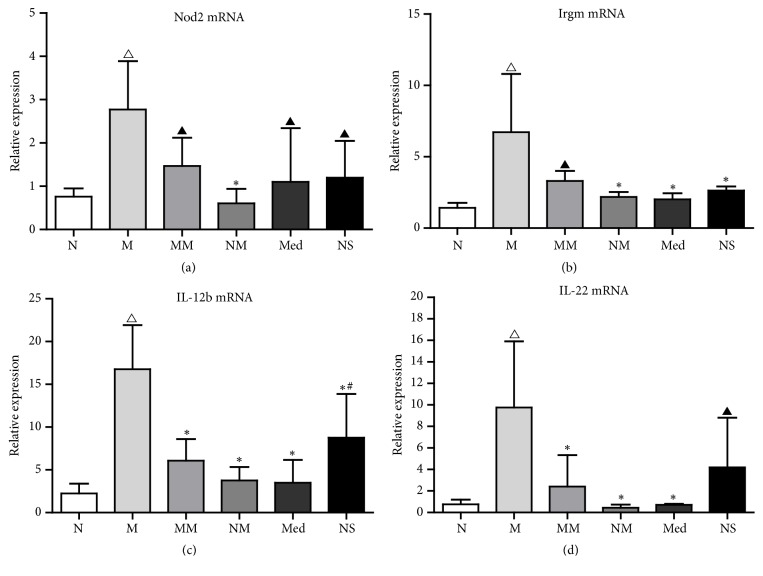
The expression of differential expression mRNA in colon tissues of rats. (a) The expression of Nod2 mRNA in rat colon tissues. (b) The expression of Irgm mRNA in rat colon tissues. (c) The expression of IL-12bmRNA in rat colon tissues. (d) The expression of IL-22 mRNA in rat colon tissues. N: normal group; M: CD model group; MM: CD model with herb-partitioned moxibustion group; NM: normal with herb-partitioned moxibustion group; Med: CD model with mesalazine group; NS: CD model with normal saline group. ^△^*P* < 0.01 versus N group; ^▲^*P* < 0.05 versus M group; ^*∗*^*P* < 0.01 versus M group; ^#^*P* < 0.05 versus Med group. P values between different groups were calculated from one-way ANOVA and LSD test.

According to the results of analysis of differential gene expression profiles, we found that the expression of* Nod2*,* Irgm*,* Il-12b*, and* Il-22* genes in the M group increased compared to the N group, and HPM and mesalazine both downregulated the expression of autophagy-associated* Nod2*,* Irgm*. Interestingly, mesalazine treatment downregulated the expression of* Il-12b*,* Il-22* genes while HPM treatment downregulated the expression of* Il-12b*,* Il-22* receptor genes of* Il-12rb1* and* Il-22ra2*. Finally we selected the Nod2, Irgm, IL-12b, IL-22 for validation using the qRT-PCR. The expression levels of Nod2, Irgm, IL-12b, and IL-22 mRNA were increased in the M group compared to the N group (*P*<0.01). While the expression of Nod2, Irgm, IL-12b, IL-22 mRNA were decreased in the MM and Med groups compared to the M group (*P*<0.01 or* P*<0.05) (Figure 6).

## 4. Discussion

CD is a chronic, intractable, intestinal disease. Currently, the western medicine treatments of CD mainly include salicylic acid preparations, hormones, immunosuppressive agents, biological agents, antibacterial agents, and probiotics. However, long-term use of western medicine will produce obvious side effects, and recurrence is frequently observed after drug withdrawal. Therefore, we need treatment methods that are effective and convenient, have limited side effects, and are accepted by patients. Previous studies showed that acupuncture and moxibustion have definite efficacy on CD; especially that HPM could attenuate abdominal pain and diarrhea in mild to moderate CD patients [13–15]. In this study, HPM significantly improved the pathological injury of colon tissues in CD rats, which was consistent with previous studies [5, 16].

The pathogenic mechanism of CD is a complex process and genetic factors play an important role in the development of CD [17]. Genome-wide association studies (GWAS) and meta-analysis showed that there are 163 IBD-associated genomes [18] and 71 CD-associated genomes [19]. In 2001, Hugot et al. [20] and Ogura et al. [21] reported that the NOD2 gene (also known as CARD) at the* OBD1* locus was the first CD-susceptibility gene in humans. Subsequently, single nucleotide polymorphisms of autophagy-associated genes of ATG16L1 [22], IRGM [23], and ULK1 [24] were found to affect autophagy and were closely associated with the development of CD [25].

Therefore, we performed RNA-Seq to observe the changes of gene expression in colon tissues of CD rats and the regulating effect of HPM. The results showed that HPM and mesalazine treatments both reduced the high expression levels of autophagy-associated genes of* Nod2*,* Irgm*, while mesalazine treatment also downregulated the* Atg9b *gene expression in colon of CD rats. The high expression levels of Nod2, Irgm mRNA in the CD model were also decreased by HPM and mesalazine treatments. NOD2 belongs to the nucleotide-binding oligomerization domain-like receptor protein family and is an intracellular pattern recognition receptor of the Nod-like receptor family [26]. ATG16L1 is a risk allele gene of CD; its single nucleotide polymorphisms (SNPs) associated with autophagy are the key factor in the autophagic response to invading pathogens and increasing the risk of CD [27]. Autophagy mediated by ATG16L1 plays an important role in the intestinal epithelium in CD. Intestinal epithelial Paneth cells exhibit notable abnormalities in the granule exocytosis pathway in ATG16L1-deficient mice, and the functional changes of many biological processes that these cells involved in directly influence intestinal injury [28]. While NOD2 can mediate autophagy and the pathological process of CD through ATG16L1, mainly in ATG16L1 can inhibit the production of inflammatory cytokines by NOD2 via its autophagy [29]. ATG9b is also an autophagy gene. The phagophores driven by ATG9b can promote the binding of LC3 and p62 to initiate autophagy-associated degradation. ATG9b deficiency can block the recruitment of p62-associated ubiquitinated protein for autophagic lysosome degradation to participate in autophagy regulation [30]. IRGM is both an autophagy gene and a risk gene of IBD. The rs13361189 and rs4958847 gene polymorphisms within* IRGM *both have important effects on the development of CD and its features of clinical phenotypes [31]. Studies found that IRGM activates core autophagy, and it can couple to innate immune receptors. Microbial products or bacterial invasion can result in increased IRGM expression and AMPK stability. Specific protein-protein interactions and the ubiquitination of IRGM can activate the interactions of IRGM and the key autophagy regulator ULK1 and the human tumor suppressor gene Beclin1 (BECN1) and promote their coassembly, thus governing the formation of autophagy initiation complexes. Furthermore, IRGM can interact with ATG16L1 and NOD2 of CD risk factors to form a molecular complex. The interaction between IRGM, NOD2 and other pattern recognition receptors such as NOD1, RIG-I, and certain TLRs can transduce microbial signals to the core autophagy apparatus to control autophagy development [32, 33].

Immune factors are also important in the pathogenic mechanism of CD [34–36]. Invasion of various pathogens and harmful microbes destroys the intestinal mucosal epithelial barrier to increase the intestinal mucosal permeability, induce overreaction of intestinal immune system, release a series of cytokines and inflammatory mediators, activate immune response, and result in the CD tissue injury, thus inducing a series of pathological changes and clinical manifestations. Therefore, in addition to autophagy-associated genes, we focused on the expression of immune-associated cytokine genes. The results showed that the expression of* Il-12b*,* Il-22 *genes in the colon tissues of CD rats were significantly increased, and mesalazine and HPM treatment respectively reduced the expression of* Il-12b*,* Il-22* genes and their receptor of* Il-12rb1* and* Il-22ra2* genes. In addition, we only selected the IL-12b and IL-22 for further validation based on the result of gene expression profile and the positive correlation between cytokines and their receptors [37]. Our results showed that the level of IL-12b and IL-22 mRNA were decreased through HPM and mesalazine treatment. IL-12 is an immune cell growth-stimulating factor in the interleukin-12 family with many biological activities. IL-12 can promote the differentiation and proliferation of T lymphocytes and NK cell, regulate cellular immunity, and increase the killing function of NK/LAK cells and the response ability of specific CTL cells [38]. IL-12b1 and IL-12b2 are the two subunits of IL-12 consisting of the functional IL12 receptor complex, and two subunits, IL-12rb1 and IL-12rb2 of the IL-12 receptor, can influence the response of T cells to IL-12. The IL-12/IL-12R signal regulates the differentiation of Th1 cell and immune response in the lesion locations of CD, thus increasing the release of Th1-type inflammatory cytokines IFN-*γ* and TNF-*α* [39]. Study found that the gene-gene interactions between the SNPs of IL-12 and IL-12 receptor increase risk of developing some disease [40], and Il-12 can also induce tyrosine phosphorylation associated with the Il-12rb1 and then have multiple immunomodulatory effects [41]. IL-22 is a member of the IL-10 family of cytokines produced by adaptive and innate immune cells; it can also be produced by activated Th1 and Th17 cells. IL-22 exerts proinflammatory effects in IBD by acting on the colonic epithelial myofibroblasts to induce the expression of proinflammatory cytokines and matrix-degrading molecules [42]. The expression of IL-22 mRNA increased in both colon and mesenteric lymph nodes in mice with colitis [43]. Besides, the level of serum IL-22 is also significantly increased and correlated with disease activity [44]. IL-22RA2 is one of the receptors of IL-22; the expression of IL-22 mRNA and IL-22RmRNA were both increased in inflammatory tissues [45]. IL-22 cannot be involved in the immunological activity of B cells due to the absence of a functional IL-22R at the surface of these cells [46]. So, in combination with our results, we speculated that the expression trend of IL-12b, IL-22 and their receptors of IL-12rb1 and IL-12rb2 may be consistent.

Additionally, it is to be noted that normal saline was used as a control in the present study. In general, intragastric administration of normal saline has no effect on intestinal inflammation [47]. However, interestingly, we found that the body weight was increased and the gene expression was changed in CD with normal saline group rats, but the trend of improvement was lower than that of herb-partitioned moxibustion and mesalazine interventions. As moxibustion improved the pathological injury and inflammation of colon in CD rats while normal saline had no such obvious effect, we speculated that although saline solution altered gene expression through some ways unclear, it may not necessarily alter the translation process of genes and then take effect. Further studies can focus on protein expression of these genes after translation to reveal the unique regulating effect of moxibustion.

Pathway enrichment analysis indicated that pathways that involved differential genes such as the CAM pathway and the MAPK pathway were associated with inflammatory reactions [48–50]. However, autophagy-associated pathways were not enriched in this study. HTLV-I basic leucine zipper factor (HBZ) that exists in the human T-cell leukemia virus type-1 (HTLV-1) infection pathway has been confirmed to regulate the activity of cellular transcription factors. In addition, HBZ can activate the mTOR mediated signaling pathways through binding to other factors [51]. In summary, herb-partitioned moxibustion might influence signaling pathways that are closely associated with CD through the regulation of autophagy- and immune-associated gene expression profiles in colon tissues of rats to effectively attenuate intestinal inflammation and promote the repair of intestinal tissue injury. These results will provide a new foundation for studies on the pathogenic mechanism of CD and the underlying mechanism of HPM treatment on CD from the perspectives of autophagy and immunology.

## Figures and Tables

**Table 1:** Differential expression of autophagy- and immune-associated genes between N group and M group. N: normal group, M: CD model group.

Upregulation		Downregulation	
Gene name	log2 Ratio	Gene name	log2 Ratio
	(M/N)		(M/N)
Il12b	6.119	Il23r	-1.322
Il22	3.858	LOC685590	-0.926
Atg9b	2.459		
Htr2b	2.225		
Il21r	2.129		
LOC103690381	1.931		
Nod2	1.357		
Tsc22d4	1.219		
Slc44a2	1.173		
Irgm	1.106		
Slc25a4	0.839		

**Table 2:** Differential expression of autophagy- and immune-associated genes between MM group and M group. MM: CD model with herb-partitioned moxibustion group, M: CD model group.

Upregulation		Downregulation	
Gene name	log2 Ratio	Gene name	log2 Ratio
	(MM/M)		(MM/M)
Rn5s	4.247	Il21	-5.714
Mmp10	4.076	Il21r	-3.368
LOC100363136	3.930	Atg9b	-2.459
Tff3	3.807	Il22ra2	-2.324
Zg16	3.705	Il12rb1	-1.067
		Nod2	-0.902
		LOC685590	-0.837
		Irgm2	-0.68
		Atg14	-0.659
		Ifnlr1	-0.62
		Il22ra1	-0.613
		Atg2a	-0.582

**Table 3:** Differential expression of autophagy- and immune-associated genes between Med group and M group. Med: CD model with mesalazine group; M: CD model group.

Upregulation		Downregulation	
Gene name	log2 Ratio	Gene name	log2 Ratio
	(Med/M)		(Med/M)
Il17a	2.907	Il22	-3.858
Il17b	1.285	Il12b	-3.119
Il23r	1.280	Tnfrsf18	-2.157
Il17rd	0.606	Tnfsf9	-1.776
		Tgfb1	-1.548
		Tgfb2	-0.996
		Il17d	-0.945
		Il12rb1	-0.884
		Tgfb3	-0.866
		Irgm	-0.84
		Il22ra2	-0.8
		Nod2	-0.771
		Tgfbr2	-0.717
		Ifnar1	-0.706
		Tgif1	-0.617

**Table 4:** Differential expression of autophagy- and immune-associated genes between NS group and M group. NS: CD model with normal saline group; M: CD model group.

Upregulation		Downregulation	
Gene name	log2 Ratio	Gene name	log2 Ratio
	(NS/M)		(NS/M)
Ifng	5.426	Atg9b	-0.874
Il27ra	2.015		
LOC685590	1.895		
Il21r	1.653		
Il21	1.216		
Il23r	1.147		
Il17b	0.736		
Tgfbr1	0.620		

## Data Availability

Data of partial gene sequencing have been presented in the article. The original data of excel format used to support the findings were supplied by Lu-Yi Wu under license and so cannot be made freely available. Requests for access to these data should be made to Lu-Yi Wu, luyitcm@163.com.

## Supplementary Materials

Supplementary Materials The complementary information in the supplementary materials is as follows. Figure S1: body weights of rats in all groups at different time points. Figure S2: heatmap of the correlation between different groups. Figure S3: upregulation and downregulation of significantly differential genes between different groups.
