# Supplementary material for: Effect of Herb-Partitioned Moxibustion on Autophagy and Immune-Associated Gene Expression Profiles in a Rat Model of Crohn's Disease
Source: Evid Based Complement Alternat Med. 2019 Mar 7;2019:3405146. doi: 10.1155/2019/3405146 (PMC6431444; doi:10.1155/2019/3405146)
Supplement: Supplementary Materials — The complementary information in the supplementary materials is as follows. Figure S1: body weights of rats in all groups at different time points. Figure S2: heatmap of the correlation between different groups. Figure S3: upregulation and downregulation of significantly differential genes between different groups. [file 3405146.f1.docx]

**Supplementary Materials**


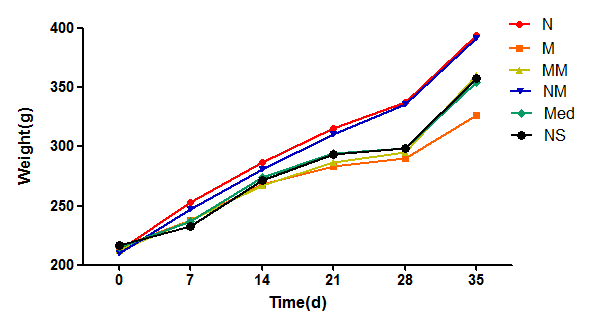


Supplementary Figure S1: Body weights of rats in all groups at different time points. N: Normal group; M: CD model group; MM: CD model with herb-partitioned moxibustion group; NM: Normal with herb-partitioned moxibustion group; Med: Mesalazine group; NS: Normal saline group.


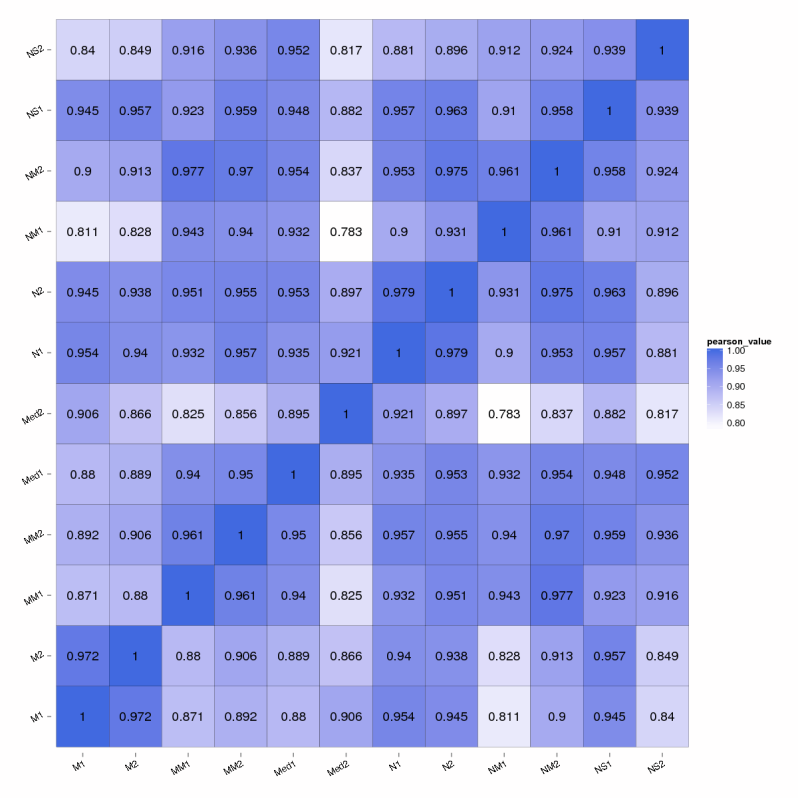


Supplementary Figure S2: Heat map of the correlation between different groups. N: Normal group; M: CD model group; MM: CD model with herb-partitioned moxibustion group; NM: Normal with herb-partitioned moxibustion group; Med: Mesalazine group; NS: Normal saline group.


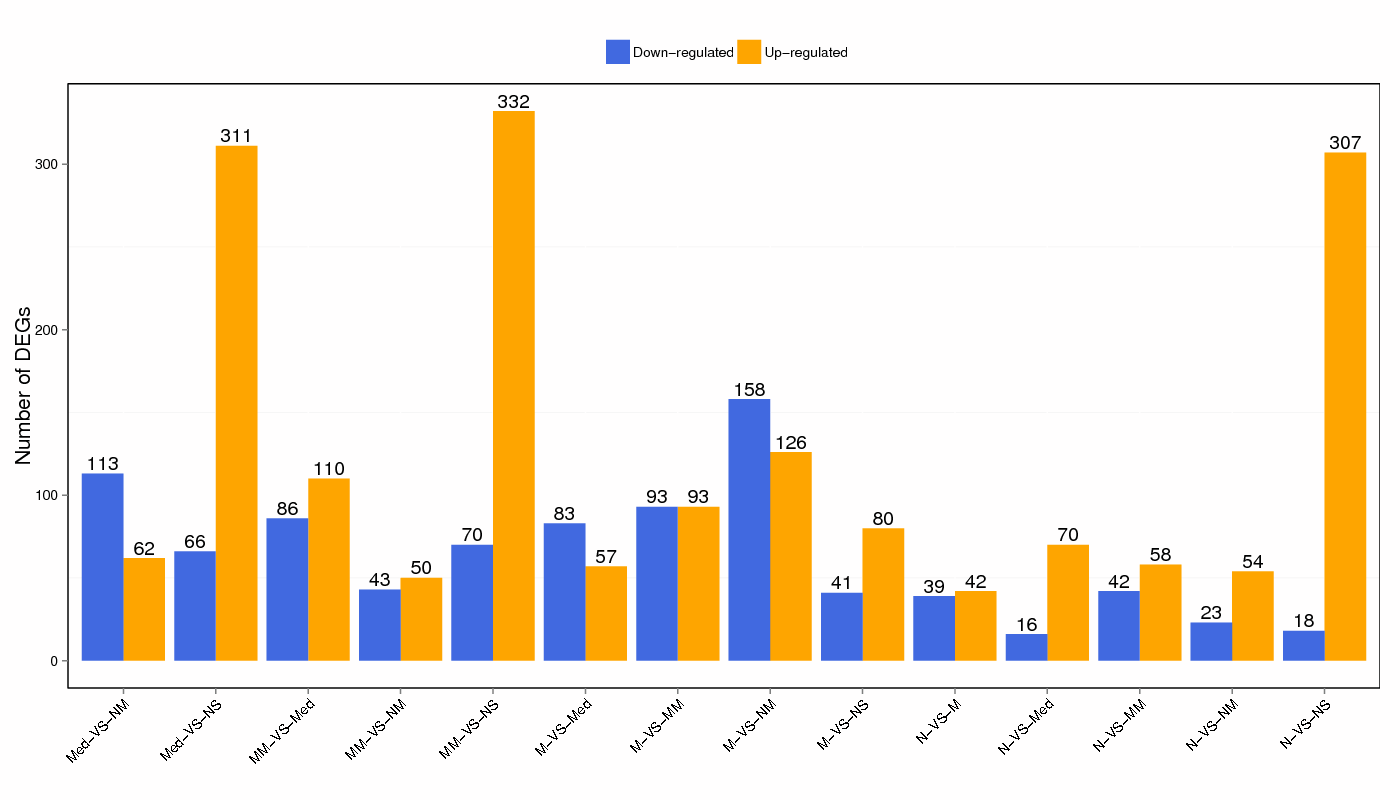


Supplementary Figure S3: Up-regulation and down-regulation of significantly differential genes between different groups. N: Normal group; M: CD model group; MM: CD model with herb-partitioned moxibustion group; NM: Normal with herb-partitioned moxibustion group; Med: Mesalazine group; NS: Normal saline group.
